# MRI changes in diaphragmatic motion and curvature in Pompe disease over time

**DOI:** 10.1007/s00330-022-08940-y

**Published:** 2022-07-13

**Authors:** Laurike Harlaar, Pierluigi Ciet, Gijs van Tulder, Harmke A. van Kooten, Nadine A. M. E. van der Beek, Esther Brusse, Marleen de Bruijne, Harm A. W. M. Tiddens, Ans T. van der Ploeg, Pieter A. van Doorn

**Affiliations:** 1 Department of Neurology, Center for Lysosomal and Metabolic Diseases, Erasmus MC, University Medical Center Rotterdam, Mailbox 2040, Rotterdam, CA 3000 The Netherlands; 2 Departments of Radiology and Nuclear Medicine and Respiratory Medicine and Allergology, Erasmus MC – Sophia Children’s Hospital, University Medical Center Rotterdam, Rotterdam, The Netherlands; 3 Department of Radiology and Nuclear Medicine, Erasmus MC, University Medical Center Rotterdam, Rotterdam, The Netherlands; 4 Department of Computer Science, University of Copenhagen, Copenhagen, Denmark; 5 Department of Paediatrics, Center for Lysosomal and Metabolic Diseases, Erasmus MC – Sophia Children’s Hospital, University Medical Center Rotterdam, Rotterdam, The Netherlands

**Keywords:** Glycogen storage disease type II, Magnetic resonance imaging, Diaphragm, Respiration, Enzyme replacement therapy

## Abstract

**Objectives:**

To evaluate changes in diaphragmatic function in Pompe disease using MRI over time, both during natural disease course and during treatment with enzyme replacement therapy (ERT).

**Methods:**

In this prospective study, 30 adult Pompe patients and 10 healthy controls underwent pulmonary function tests and spirometry-controlled MRI twice, with an interval of 1 year. In the sagittal view of 3D gradient echo breath-hold acquisitions, diaphragmatic motion (cranial-caudal ratio between end-inspiration and end-expiration) and curvature (diaphragm height and area ratio) were calculated using a machine learning algorithm based on convolutional neural networks. Changes in outcomes after 1 year were compared between Pompe patients and healthy controls using the Mann-Whitney test.

**Results:**

Pulmonary function outcomes and cranial-caudal ratio in Pompe patients did not change significantly over time compared to healthy controls. Diaphragm height ratio increased by 0.04 (−0.38 to 1.79) in Pompe patients compared to −0.02 (−0.18 to 0.25) in healthy controls (*p* = 0.02). An increased diaphragmatic curvature over time was observed in particular in untreated Pompe patients (*p* = 0.03), in those receiving ERT already for over 3 years (*p* = 0.03), and when severe diaphragmatic weakness was found on the initial MRI (*p* = 0.01); no progression was observed in Pompe patients who started ERT less than 3 years ago and in Pompe patients with mild diaphragmatic weakness on their initial MRI.

**Conclusions:**

MRI enables to detect small changes in diaphragmatic curvature over 1-year time in Pompe patients. It also showed that once severe diaphragmatic weakness has occurred, improvement of diaphragmatic muscle function seems unlikely.

**Key Points:**

• *Changes in diaphragmatic curvature in Pompe patients over time assessed with 3D MRI may serve as an outcome measure to evaluate the effect of treatment on diaphragmatic function*.

• *Diaphragmatic curvature showed a significant deterioration after 1 year in Pompe patients compared to healthy controls, but the curvature seems to remain stable over this period in patients who were treated with enzyme replacement therapy for less than 3 years, possibly indicating a positive effect of ERT*.

• *Improvement of diaphragmatic curvature over time is rarely seen in Pompe patients once diaphragmatic motion shows severe impairment (cranial-caudal inspiratory/expiratory ratio < 1.4)*.

**Supplementary Information:**

The online version contains supplementary material available at 10.1007/s00330-022-08940-y.

## Introduction

Pompe disease is a genetic metabolic disorder caused by the deficiency of the lysosomal enzyme alpha-glucosidase which leads to glycogen accumulation in many tissues, particularly muscles. In adults, it usually manifests as a limb girdle myopathy with respiratory insufficiency [1]. Due to predominant diaphragmatic weakness, respiratory insufficiency is especially present in the supine position and therefore nocturnal mechanical ventilation is needed in 32–80% of adult patients [2–6].

Monitoring of respiratory muscle weakness is important to evaluate the need for nocturnal mechanical ventilation and to assess treatment effects. Currently, enzyme replacement therapy (ERT) with recombinant human acid α-glucosidase (alglucosidase alfa, myozyme®) is the only approved therapy for adult Pompe patients, but next-generation enzyme replacement therapies and gene therapies are being developed [7–9]. Surprisingly, the effect of ERT on respiratory muscles seems smaller than the effect on skeletal muscle function [10–13]. At group level, pulmonary function outcomes after 5–10 years of ERT were better than the expected natural course. Individuals however may show progressive respiratory weakness despite start of ERT or may have an initial positive effect followed by a secondary decline [5, 11, 13]. The pathophysiological background of the progressive pulmonary dysfunction in these patients is currently unknown.

Pulmonary function tests are used to evaluate vital capacity or forced vital capacity (FVC) in upright seated and supine position, and to assess respiratory muscle strength by using maximum inspiratory and expiratory mouth pressures (MIP and MEP) [10]. A drawback of these tests is that they do not provide specific insight into the function of the individual muscles involved in respiration. Using MRI, it is possible to study diaphragmatic function and thoracic movements separately. In cross-sectional MRI studies in Pompe patients, it was shown that motion of the diaphragm was decreased while motion of the thoracic wall was normal [14–16]. In healthy controls, the curvature of the diaphragm during inspiration was decreasing, while in Pompe patients, the curvature was increasing, indicating insufficient diaphragmatic contraction. Importantly, early changes in diaphragmatic motion could already be observed while FVC was still within the normal range [17].

The purpose of this study was to evaluate changes in diaphragmatic function over time in patients with Pompe disease using MRI. Pompe patients and healthy controls were studied twice, with an interval of 1 year, using a standardized MRI protocol with automatic machine learning–derived imaging analysis techniques.

## Materials and methods

### Study design

This prospective cohort study was performed at the Center for Lysosomal and Metabolic Diseases at Erasmus University Medical Center, the reference center for Pompe disease in The Netherlands. In the initial cross-sectional study, 35 patients with Pompe disease and 18 gender- and age-matched healthy controls were included [17]. Inclusion criteria were a confirmed diagnosis of non-classic Pompe disease (based on two disease-causing variants in the acid alpha-glucosidase gene and/or decreased enzyme activity in fibroblasts) and the ability to lie in a supine position for at least 30 min without mechanical ventilation. Exclusion criteria were comorbidities or devices that did not permit MRI investigations, and claustrophobia. For the current prospective study, all Pompe patients and 10 of the healthy controls were invited to repeat the study procedures after 1 year, comprising regular pulmonary function tests and spirometry-controlled MRI scans using a 20–25-min MRI protocol [17]. Of the 35 Pompe patients that took part in the initial cross-sectional study, five patients could not be included in this follow-up study for the following reasons: living abroad (*n* = 1), withdrawn participation (*n* = 2), or participation in studies using other treatment (*n* = 2). All study procedures were performed between January 2016 and February 2019. The protocol was approved by the Medical Ethical Committee at our hospital (MEC-2007-103, amendment 7) and all participants provided written informed consent.

### Pulmonary function tests

All study procedures were performed on 1 day in the same order, starting with pulmonary function tests. Pulmonary function tests comprised FVC in upright seated and supine position and MIP and MEP in upright seated position. All tests were performed according to American Thoracic Society/European Respiratory Society standards and results were expressed as a percentage of predicted normal values [18–20]. During pulmonary function tests, participants were trained to perform specific breathing maneuvers during MRI scanning. The MRI was always obtained directly after this training.

### MRI analysis

All participants were examined on a 3-T Signa 750 MRI scanner (General Electric Healthcare) using the whole-body coil for radiofrequency excitation and a 32-channel torso coil for signal reception [17]. In the current study, we used three-dimensional (3D) spoiled-gradient echo (SPGR) breath-hold acquisitions (repetition time/echo time = 1.2/0.5 ms, flip angle 2°, voxel resolution 3 × 3 × 3 mm) at end-inspiration and end-expiration. Breathing maneuvers during MRI were standardized using an MR-compatible spirometer to ensure the best performance of each subject for inspiratory and expiratory scans [21]. The maximum breath-hold time was 10 s and scans were only repeated if the image quality was poor.

**Fig. 1:**
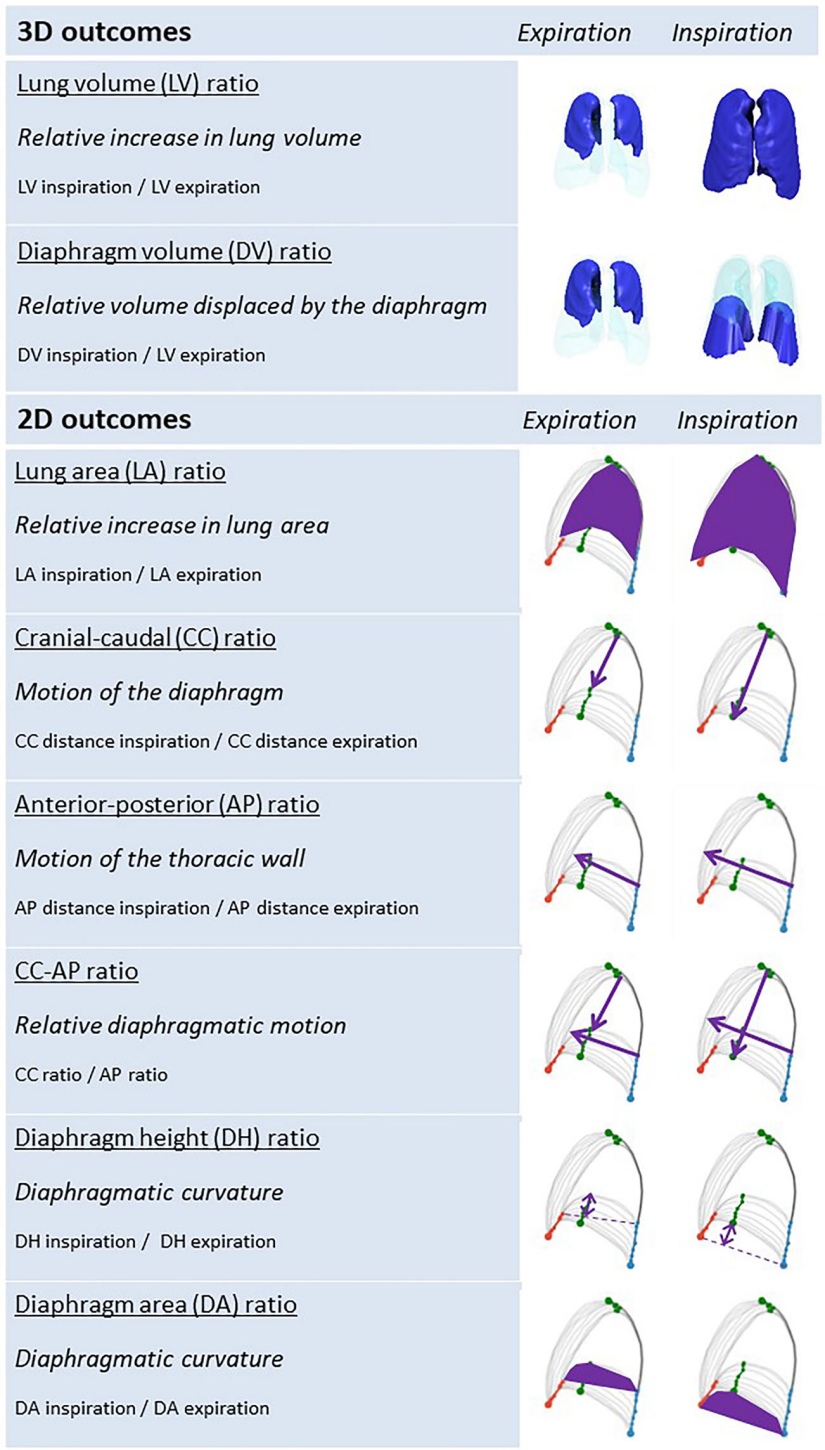
**Outcome measures**. Examples of 3D outcomes and 2D outcomes in the sagittal view at the right mid hemi-diaphragm of 3D breath-hold scans. 2D outcomes were obtained from previously conducted dynamic 2D imaging studies [17]. For all outcomes, we calculated a ratio between end-expiration and end-inspiration outcomes to adjust for differences in sex, age, and size of participants

For image analysis, we developed automatic lung segmentations using a machine learning algorithm based on convolutional neural networks [22]. In these segmentations, we calculated 3D outcomes [1–2] and two-dimensional (2D) outcomes [3–8]: (1) the lung volume ratio to evaluate the relative increase in lung volume from end-expiration to end-inspiration, (2) the diaphragm volume ratio to evaluate the relative volume displaced by the diaphragm, (3) the lung area ratio to evaluate the relative increase in the lung area, (4) the cranial-caudal ratio to evaluate the motion of the diaphragm, (5) the anterior-posterior ratio to evaluate the motion of the thoracic wall, (6) the relative cranial-caudal ratio compared to the anterior-posterior ratio (CC-AP ratio) to evaluate the diaphragm motion relative to motion of the thoracic wall, and (7) the diaphragm height ratio and (8) the diaphragm area ratio to evaluate the diaphragmatic curvature (Fig. 1). Previously, we obtained 2D outcomes from 2D dynamic images [17]. In the current study, analysis of the 2D outcomes was obtained from the sagittal view at the right mid hemi-diaphragm of 3D breath-hold scans. Based on image registration of the 3D scans, the sagittal levels of the initial and follow-up MRI were automatically matched. For all MRI outcomes, we used a ratio between inspiration and expiration to adjust for possible differences in age, sex, height, and weight of the patients. All outcomes were automatically calculated using in-house developed software in Python (Python 3.6.3, https://www.python.org/, ©2001–2019; Python Software Foundation; SciPy 1.1.0, https://www.scipy.org/, ©2003–2019 SciPy developers) for the 3D segmentation and 2D outcomes, with postprocessing in MATLAB (MathWorks) for the 3D outcomes. All automatic segmentations and measurements were manually checked, and segmentations were corrected if necessary. A detailed description of the procedures is presented in the supplementary material.

### Statistical analysis

Differences in demographics (sex, age, height, weight, BMI), pulmonary function test outcomes, and MRI outcomes between Pompe patients and healthy controls were analyzed using the chi-square test for sex and the Mann-Whitney test for the other variables. To calculate changes over time in pulmonary function test outcomes and MRI outcomes, we calculated the differences between follow-up and initial results.

To investigate the possible effect of ERT on changes over time in MRI outcomes, we analyzed subgroups of (1) patients without ERT, (2) patients with ≤ 3 years ERT at the initial MRI, and (3) patients with > 3 years ERT at the initial MRI, based on the peak clinical effect of ERT that is usually reached in the first 3 years of treatment [11, 13]. Moreover, we compared subgroups of patients with no or minor diaphragmatic weakness at the initial MRI (defined as a cranial-caudal ratio ≥ 1.4) and patients with moderate or severe diaphragmatic weakness (cranial-caudal ratio < 1.4). This cut-off of 1.4 was chosen on the basis of the lowest cranial-caudal ratio recorded in healthy controls [17]. Differences between each subgroup and healthy controls were tested using the Mann-Whitney test. Because subgroups were small and these analyses are indicative only, we did not correct for multiple testing. Statistical analysis was performed with SPSS for Windows (version 24, SPSS Inc). The significance level was set at *p* ≤ 0.05.

## Results

### Participant characteristics

We included 30 Pompe patients (14 men, median age 37 years (range 18–70), 16 women, median age 44 years (range 17–70)) and 10 healthy controls (5 men, median age 32 years (range 26–61), 5 women, median age 42 years (range 25–62)) (Table 1). There were no significant differences in sex, age, height, and weight between Pompe patients and healthy controls. One Pompe patient used nocturnal mechanical ventilation and none of the patients used a wheelchair. Median time between initial and follow-up MRI was longer in healthy controls than in Pompe patients (1.13 vs 1.02 years, *p* = 0.018). Individual outcomes in each subject are presented in the supplementary material.

**Table 1:** Baseline characteristics

	Pompe patients (*n* = 30)	Healthy controls (*n* = 10)	*p* value
Sex, *number of males (%)*	14 (47%)	5 (50%)	0.855
Age, *years*	43 (17–70)	37 (26–63)	0.685
Height,* cm*	174 (157–201)	180 (165–194)	0.617
Weight, *kg*	71 (56–99)	76 (55–99)	0.542
BMI, *kg/m*^*2*^	23 (19–34)	23 (20–27)	0.779
Disease duration, *years*	12 (0–38)	-	-
Patients without ERT, *n* (%)	7 (23%)	-	-
Patients ≤ 3 years ERT, *n* (%) *ERT duration at initial measurement, years*	8 (27%) 0.65 (−0.1 to 3.0)	-	-
Patients > 3 years ERT, *n* (%) *ERT duration at initial measurement, years*	15 (50%) 9.2 (3.7–12.8)	-	-
Follow-up time between follow-up and initial measurement, *years*	1.02 (0.88–2.01)	1.13 (1.0–1.63)	0.018*

Characteristics of Pompe patients and healthy controls. Continuous values are presented as median (min – max) and tested with the Mann-Whitney test, categorical values as number with percentage and tested with the chi-square test. *BMI* body mass index, *ERT* enzyme replacement therapy. Significant differences are indicated by asterisks (*)

### Pulmonary function test outcomes

Pompe patients had lower values of pulmonary function test results than healthy controls at the initial measurement (Table 2). The changes in FVC upright and supine, MIP, and MEP after 1 year were not significantly different between Pompe patients and healthy controls (Table 2).

**Table 2:** Pulmonary function test outcomes

	Initial measurement			Difference between follow-up and initial measurement		
	Pompe patients	Healthy controls	*p* value	Pompe patients	Healthy controls	*p* value
FVC upright	91 (67–119)	106 (96–114)	< 0.001*	−3.4 (−21.1 to 4.3)	−1.6 (−4.7 to 0.14)	0.303
FVC supine	71 (29–115)	104 (93–114)	< 0.001*	−2.3 (−14.8 to 3.8)	−2.7 (−8.6 to 2.4)	0.639
Δ FVC	15 (−2 to 38)	3 (−1 to 8)	0.004*	−0.9 (−8.6 to 6.6)	0.7 (−5.0 to 8.0)	0.248
MIP	71 (37–127)	102 (54–131)	0.041*	3.9 (−26.6 to 50.5)	5.0 (−18.5 to 28.6)	0.950
MEP	85 (35–149)	102 (67–132)	0.042*	0.3 (−29.2 to 26.7)	−4.3 (−21.3 to 5.2)	0.080

Pulmonary function test outcomes at the initial measurement and differences after 1-year follow-up in Pompe patients compared to healthy controls. Significant differences are indicated by asterisks (*). All values are expressed in % of predicted values, median (min – max). *FVC* forced vital capacity, *Δ FVC* FVC upright − FVC supine, *MIP* maximum inspiratory pressure, *MEP* maximum expiratory pressure

### MRI outcomes

**Fig. 2 Examples of 3D MRI images.**
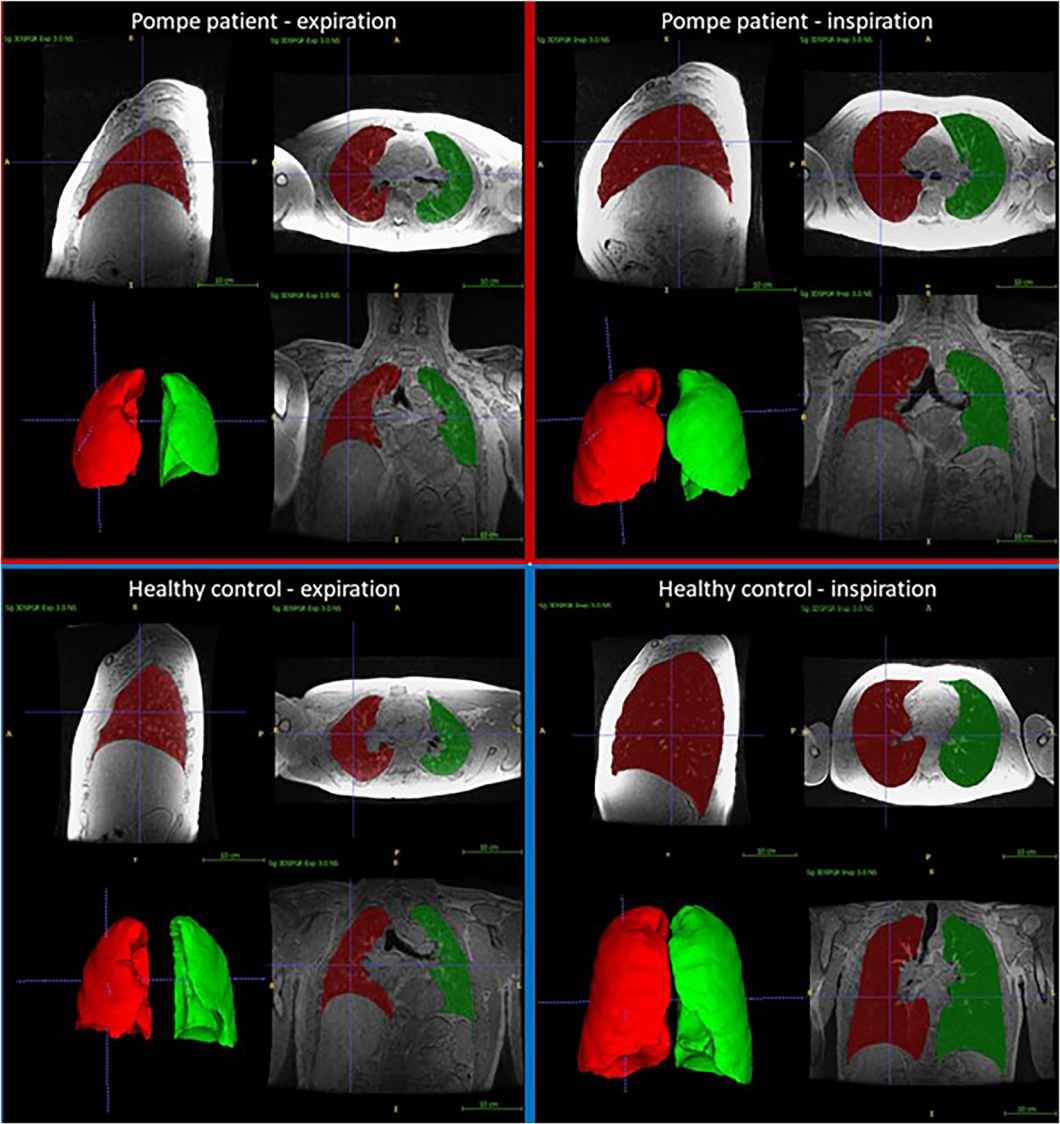
Example of 3D breath-hold scans at end-expiration and end-inspiration in a Pompe patient and in a healthy control, including automatic segmentations of the left lung (green) and the right lung (red). This Pompe patient has a decreased motion of the diaphragm, decreased volume displaced by the diaphragm, and an increased curvature of the diaphragm during inspiration

Lung volume ratio, diaphragm volume ratio, lung area ratio, cranial-caudal ratio, and CC-AP ratio at the initial MRI were lower in Pompe patients than in healthy controls (*p* < 0.01 for all outcomes) (Table 3, Fig. 2). Changes in lung volume ratio, diaphragm volume ratio, lung area ratio, cranial-caudal ratio, anterior-posterior ratio, and CC-AP ratio after 1-year follow-up were not significantly different between Pompe patients and healthy controls, indicating no change in overall lung volume or diaphragmatic motion over 1-year time.

**Table 3:** MRI outcomes

	Initial measurement			Difference between follow-up and initial measurement		
	Pompe patients	Healthy controls	*p* value	Pompe patients	Healthy controls	*p* value
Lung volume ratio	2.29 (1.27–3.58)	2.83 (2.28–3.84)	0.005*	−0.03 (−0.29 to 0.12)	−0.03 (−0.26 to 0.21)	0.803
Diaphragm volume ratio	0.41 (0.07–1.12)	0.88 (0.50–1.11)	0.002*	−0.01 (−0.15 to 0.11)	−0.01 (−0.17 to 0.15)	0.901
Lung area ratio	2.18 (1.12–3.06)	2.55 (1.88–3.12)	0.003*	−0.03 (−0.27 to 0.14)	−0.01 (−0.23 to 0.21)	0.827
Cranial-caudal ratio	1.42 (0.94–1.78)	1.72 (1.48–1.90)	< 0.001*	−0.01 (−0.11 to 0.12)	−0.02 (−0.08 to 0.16)	0.851
Anterior-posterior ratio	1.26 (1.10–1.52)	1.30 (1.16–1.48)	0.417	−0.00 (−0.06 to 0.33)	−0.02 (−0.09 to 0.05)	0.169
CC-AP ratio	1.09 (0.82–1.60)	1.31 (1.10–1.48)	0.001*	−0.01 (−0.20 to 0.11)	0.02 (−0.11 to 0.14)	0.126
Diaphragm height ratio	1.19 (0.54–2.78)	1.12 (0.75–1.72)	0.098	0.04 (−0.38 to 1.79)	−0.02 (−0.18 to 0.25)	0.019*
Diaphragm area ratio	1.48 (0.55–3.69)	1.35 (0.86–2.60)	0.248	0.04 (−0.28 to 4.40)	−0.07 (−0.27 to 0.39)	0.034*

MRI outcomes at the initial measurement and differences after 1-year follow-up in Pompe patients compared to healthy controls. Significant differences are indicated by an asterisk (*). All values are ratios between inspiration and expiration, median (min–max). *CC-AP*, cranial-caudal/anterior-posterior ratio

Diaphragm height ratio and diaphragm area ratio at the initial MRI were not significantly different between Pompe patients and healthy controls (Table 3). Interestingly, the median change in diaphragm height ratio after 1 year was 0.04 (range −0.38 to 1.79) in Pompe patients versus −0.02 (range −0.18 to 0.25) in healthy controls (*p* = 0.019) and the median change in diaphragm area ratio was 0.04 (range −0.28 to 4.40) in Pompe patients versus −0.07 (range −0.27 to 0.39) in healthy controls (*p* = 0.034). If we assume that the change in diaphragm height and area ratio after 1 year in all healthy controls was 0, the difference between Pompe patients and healthy controls in the change in diaphragm height ratio remained significant (*p* = 0.012), but the change in diaphragm area ratio was not significant (*p* = 0.208). The increasing ratios in Pompe patients indicate an increased diaphragmatic curvature after 1 year. This corresponds to a more insufficient diaphragmatic contraction and may indicate progressive diaphragmatic dysfunction.

In one Pompe patient (treated > 3 years with ERT), the increase in diaphragm height and area ratio (1.79 and 4.40) was outside the range of the results of other patients. FVC upright decreased 4% and FVC supine remained stable. The MRI results were manually checked, and not caused by an error in automatic measurements; therefore, this patient was not excluded from analysis. However, if we would have excluded this particular patient, the median change in diaphragm height ratio (0.03 (range −0.38 to 0.42), *p* = 0.024) and diaphragm area ratio (0.03 (range −0.28 to 0.49), *p* = 0.043) in Pompe patients remained significant compared to healthy controls.

### Subgroup analysis

**Fig. 3 Diaphragmatic curvature changes after 1 year related to ERT duration.**
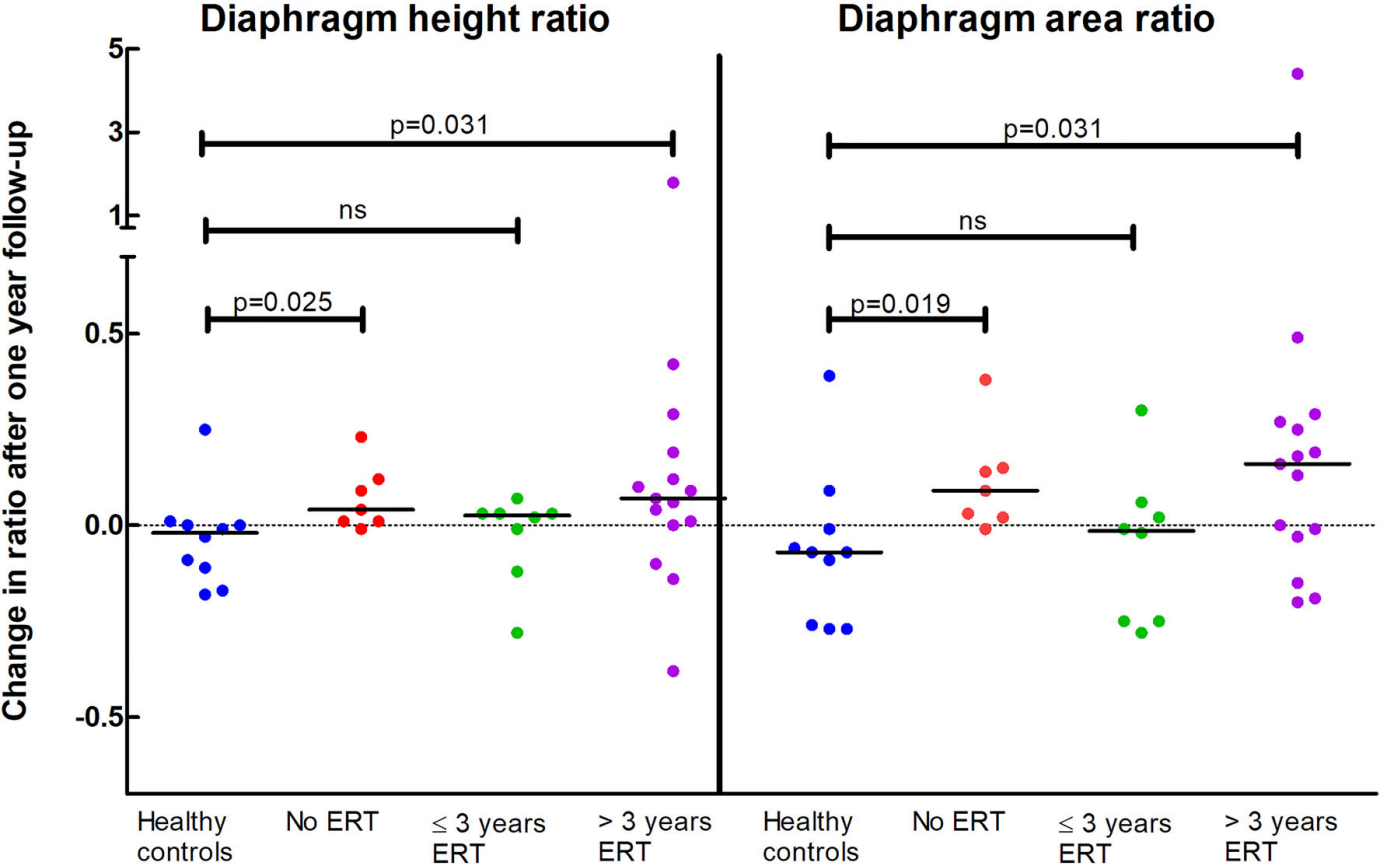
Changes in diaphragm curvature after 1-year follow-up compared to the initial MRI in subgroups of Pompe patients related to ERT duration and compared to healthy controls. An increasing diaphragm height and area ratio after 1-year follow-up (values > 0) correspond to an increasing diaphragm curvature ratio indicating a deterioration of diaphragm function over time; decreasing ratios indicate improvement. ERT, enzyme replacement therapy; ns, not significant

**Fig. 4 Examples of change of diaphragm curvature over time.**
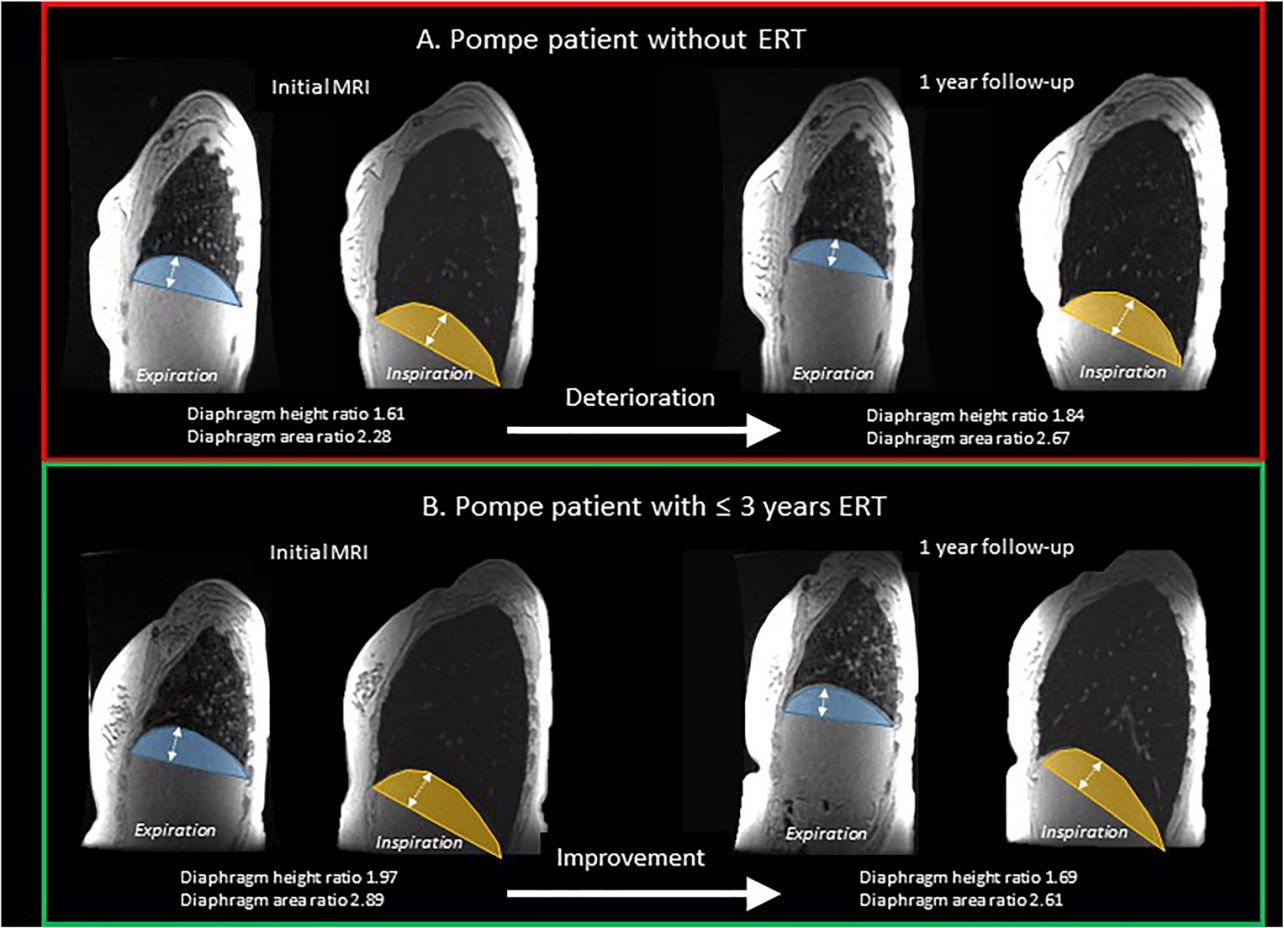
The diaphragm height is shown in white arrows. The diaphragm area is shown in blue (expiration) or yellow (inspiration). Ratios are outcomes at inspiration divided by outcomes at expiration. ERT, enzyme replacement therapy. Patient A is a female Pompe patient of 17 years old, not treated with ERT because she is asymptomatic and stable over time. At the initial MRI, both diaphragm height ratio and diaphragm ratio are increasing during inspiration (ratio > 1), indicating an insufficient diaphragmatic contraction. After 1-year follow-up, the ratios are increasing, corresponding to a deterioration of diaphragmatic curvature. At the initial measurement, forced vital capacity (FVC) upright was 93% predicted and FVC supine 95%, at follow-up FVC upright was 94% predicted and FVC supine 89%. Patient B is a female Pompe patient of 25 years old and 2.5 years treated with ERT at the initial MRI. FVC upright was 119% predicted and FVC supine 115%. The diaphragmatic curvature is increasing during inspiration (diaphragm height ratio and diaphragm ratio > 1), indicating an insufficient diaphragmatic contraction. After 1-year follow-up, FVC upright was 115% predicted and FVC supine 113%, but the diaphragmatic height and area ratios are decreased, corresponding to an improved diaphragmatic curvature

Diaphragm curvature significantly increased in non-treated Pompe patients (median change in diaphragm height ratio 0.04 (range −0.01 to 0.23), *p* = 0.025), and also in Pompe patients treated > 3 years with ERT (median change in diaphragm height ratio 0.07 (range −0.38 to 1.79), *p* = 0.031). The curvature remained stable in healthy controls and in Pompe patients treated ≤ 3 years with ERT (median change in diaphragm height ratio 0.02 (range −0.28 to 0.07), *p* > 0.05). Results of the change in diaphragm area ratio were similar to the change in diaphragm height ratio (Figs. 3 and 4).

We investigated whether the changes in diaphragmatic curvature are related to diaphragmatic weakness at the initial MRI. We found an increased diaphragmatic curvature after 1-year follow-up in Pompe patients with moderate or severe diaphragmatic weakness (cranial-caudal ratio < 1.4 at the initial MRI, *n* = 14), as the diaphragm height ratio increased with 0.03 (range −0.38 to 0.29) and the diaphragm area ratio with 0.04 (range −0.25 to 0.29) compared to healthy controls (*p* = 0.014 and *p* = 0.04). However, in Pompe patients with no or only minor diaphragmatic weakness (a cranial-caudal ratio ≥ 1.4 at the initial MRI, *n* = 16), the diaphragmatic curvature did not change significantly over time compared to healthy controls: median change in diaphragm height ratio 0.05 (range −0.28 to 1.79) and diaphragm area ratio 0.06 (range −0.28 to 4.40), both *p* = 0.082.

**Fig. 5 Change of diaphragmatic curvature after 1 year related to initial diaphragmatic weakness.**
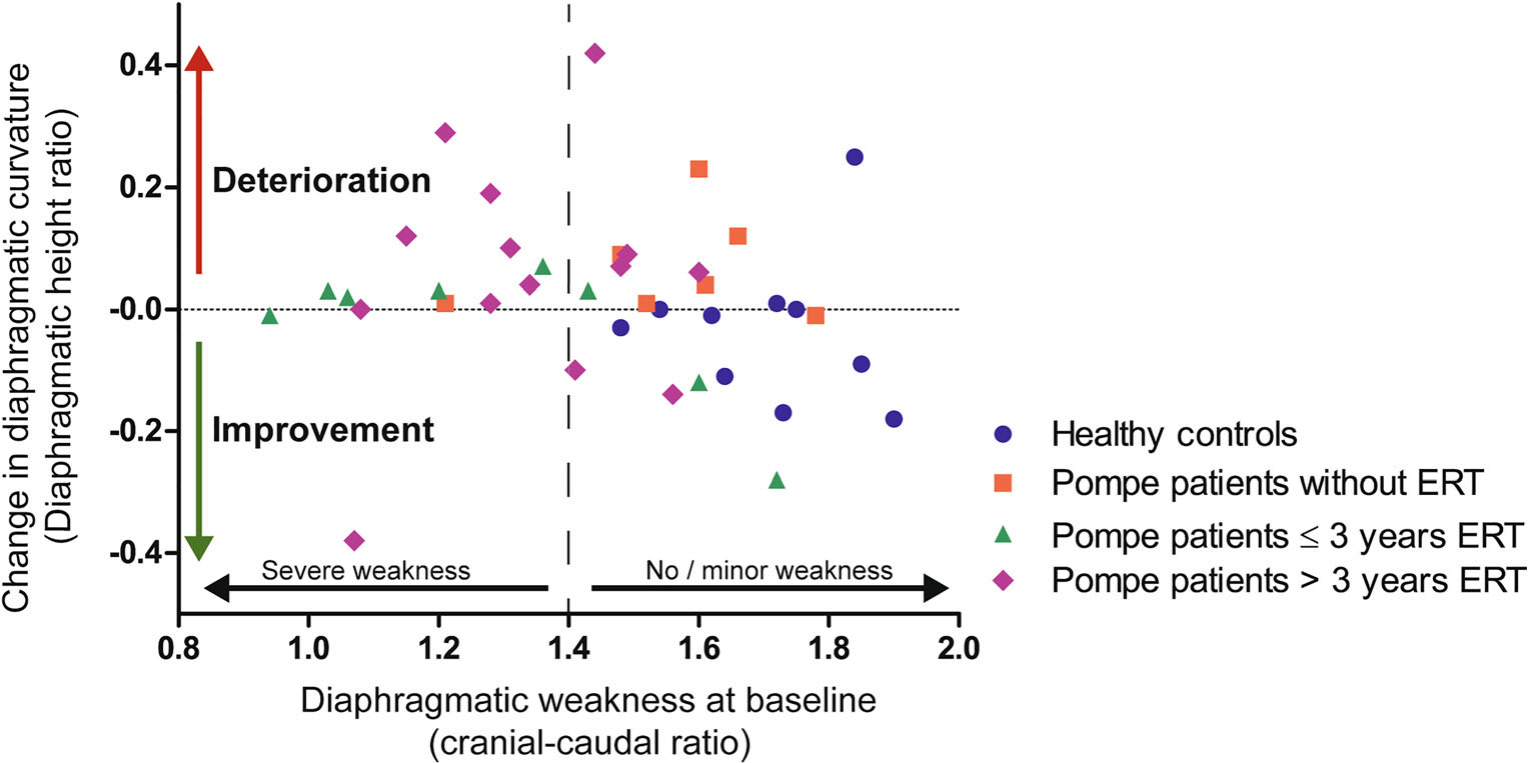
A positive change in diaphragmatic height ratio (increased diaphragmatic curvature over time) is classified as deterioration. A negative change in diaphragmatic height ratio (decreased diaphragmatic curvature over time) is classified as improvement. A cranial-caudal ratio < 1.4 is classified as moderate to severe diaphragmatic weakness at the initial MRI, and a cranial-caudal ratio ≥ 1.4 is classified as mild to no diaphragmatic weakness at the initial MRI. One Pompe patient (cranial-caudal ratio 1.48, change in diaphragmatic height ratio 1.79, > 3 years ERT) is not included in this figure because these results were outside the range of the results of other patients. ERT, enzyme replacement therapy

We investigated a possible effect of ERT on the change in diaphragmatic curvature in relation to the level of diaphragmatic weakness at the initial MRI (Fig. 5). Noteworthy, we found that two out of eight Pompe patients with a normal cranial-caudal ratio (≥ 1.4) at the initial MRI (suggesting no or minor weakness of the diaphragm), who were treated ≤ 3 years with ERT, showed a clear decrease of diaphragmatic curvature, indicating improvement of diaphragmatic function. This is in contrast to the absence of improvement of diaphragmatic curvature over time, observed in all seven Pompe patients who did not receive ERT, six of whom also had a cranial-caudal ratio ≥ 1.4 at the initial MRI. Of all Pompe patients with a cranial-caudal ratio < 1.4 at the initial MRI, indicating moderate to severe weakness of the diaphragm, only one patient showed a clear improvement of the diaphragmatic curvature over time. This particular female patient aged 68 years, was yet treated over 10 years with ERT, had mild limb girdle weakness and a fairly stable FVC upright (86%) and FVC supine (41%) over the last years.

## Discussion

Pompe patients may have progressive pulmonary dysfunction despite treatment with ERT. Changes in diaphragmatic function are difficult to detect with standard pulmonary function tests. This is the first study using MRI to evaluate diaphragmatic function in Pompe patients over time. We showed that the curvature of the diaphragm during inspiration may increase in Pompe patients already after 1-year follow-up, while standard pulmonary function tests did not change. Interestingly, the diaphragmatic curvature seems to remain stable in Pompe patients who were treated with ERT for less than 3 years, suggesting a positive effect on diaphragmatic function in the first years after starting ERT. Additionally, we found that Pompe patients with a severely decreased motion of the diaphragm, despite ERT, rarely show improvement of the diaphragmatic curvature over time.

Our current MRI protocol enabled simultaneous evaluation of both the entire diaphragm and the thoracic wall during dynamic inspiration with a relatively large field of view. For this study, we developed an advanced automatic segmentation technique and all outcome measures were calculated automatically and thus are operator independent. By using 3D MRI sequences, we were able to compare MRI outcomes on a matching sagittal level, leading to a more robust analysis.

The curvature of the diaphragm during inspiration, evaluated using diaphragm height and area ratio, increased in Pompe patients over a 1-year period. Changes in diaphragmatic curvature after 1 year are small, which could be expected because Pompe disease is a slowly progressive disorder. Because the changes in Pompe patients were significantly larger than in healthy controls, we concluded that these minor changes after 1 year are possibly an early sign of progressive diaphragmatic weakness. In contrast, the change in other MRI outcomes and pulmonary function test outcomes were not significantly different between Pompe patients and healthy controls. Therefore, we assume that the curvature of the diaphragm may increase before the obvious changes in the cranial-caudal ratio and pulmonary function tests can be observed. The increased curvature during inspiration in Pompe patients is likely caused by weakness of the diaphragm, due to insufficient muscle contraction. Because Pompe patients also have abdominal muscle weakness, abdominal pressure will insufficiently increase during inspiration and diaphragmatic weakness is not causing a paradoxical motion but only a paradoxical increased curvature [17, 23].

At the initial MRI, diaphragm height and area ratio were not significantly different between Pompe patients and healthy controls. This might be explained by a large range of disease severity between Pompe patients we evaluated, including six patients (20%) without an indication for ERT because these patients had no or only very mild symptoms of muscle weakness. However, in Pompe patients with impaired pulmonary function, the diaphragm height and area ratio were decreased compared to healthy controls [17].

An important question is whether an early start of ERT can prevent irreversible damage to the diaphragm in Pompe patients. We showed that the diaphragmatic curvature in Pompe patients with no or limited impairment of the diaphragm seems to remain stable or even improved after 1 year in patients treated with ERT for a period of less than 3 years. No improvement of the diaphragmatic curvature was observed when patients already have severe dysfunction of the diaphragm. Despite subgroups were small and these findings are indicative only, they parallel previous studies on the effects of ERT on FVC that showed that the main effects of ERT could be achieved during the first years after start of treatment [5, 11, 13]. It would also be interesting to study diaphragmatic involvement and response to ERT in subgroups of children with classic and non-classic Pompe disease.

Our study has some limitations. First, the number of participants was relatively small and patients had a large range of disease and treatment duration. In addition, Pompe patients with severe pulmonary dysfunction are not included because they are not able to lay flat without ventilation. Second, we were only able to compare one initial measurement with one follow-up measurement in each single patient. However, breathing maneuvers were standardized using an MR-compatible spirometer to improve the reliability of our measurements. As the mean follow-up time in healthy controls was longer, this may have even resulted in an underestimation of the differences between Pompe patients and healthy controls. Third, we used breath-hold scans, which may be different from the clinical situation during normal breathing. The use of dynamic 3D acquisitions potentially would be preferable; however, analysis of these images appeared to be more difficult due to a lower spatial resolution. Finally, automatic algorithms are not yet widely available, currently hampering its application in daily practice.

In conclusion, using 3D MRI, small changes in diaphragmatic curvature over time could be detected in patients with Pompe disease. Once severe diaphragmatic weakness has occurred, it is unlikely that this can improve. MRI potentially can be used to identify Pompe patients with still normal standard pulmonary function tests and only minor diaphragmatic abnormalities who might benefit from an early start of ERT. Our MRI outcome measures need further validation in larger cohort studies with a longer follow-up interval before these can be introduced as new MRI markers in clinical trials to evaluate changes in diaphragmatic function over time.

## Supplementary Information

ESM 1 (DOCX 1501 kb)

## Abbreviations

2D: Two-dimensional
3D: Three-dimensional
CC-AP ratio: Cranial-caudal ratio compared to the anterior-posterior ratio
ERT: Enzyme replacement therapy
FVC: Forced vital capacity
MEP: Maximum expiratory pressure
MIP: Maximum inspiratory pressure
SPGR: Spoiled-gradient echo

## Declarations

### Guarantor

The scientific guarantor of this publication is Prof. Pieter A. van Doorn, MD PhD.

### Statistics and biometry

Prof. Dimitris Rizopoulos (Erasmus MC, University Medical Center Rotterdam, Department of Biostatistics) kindly provided statistical advice for this manuscript.

### Study subjects or cohorts overlap

In the current prospective study, 30 Pompe patients and 10 healthy controls were included. All these participants initially participated in a cross-sectional study, in which we reported a decreased motion and an abnormal curvature of the diaphragm during inspiration in Pompe patients (Harlaar L, Ciet P, van Tulder G, et al Chest MRI to diagnose early diaphragmatic weakness in Pompe disease. *Orphanet Journal of Rare Diseases*. 2021;16(1):21).

In this follow-up study, we repeated the MRI study after 1 year, to evaluate possible changes in the motion and curvature of the diaphragm over time and during treatment with enzyme replacement therapy. We used 3D SPGR breath-hold acquisitions at end-expiration and end-inspiration, instead of dynamic sagittal 2D images that were used in the cross-sectional study. Therefore, we were able to compare MRI outcomes on a matching sagittal level at the initial MRI and the follow-up MRI. In addition, we developed automatic lung segmentations, while in the cross-sectional study we used manual lung segmentations.

### Methodology

• prospective

• case-control study

• performed at one institution
