# Supplementary material for: MRI changes in diaphragmatic motion and curvature in Pompe disease over time
Source: Eur Radiol. 2022 Jul 13;32(12):8681–91. doi: 10.1007/s00330-022-08940-y (PMC9705445; doi:10.1007/s00330-022-08940-y)
Supplement: Supplementary file 1 — (DOCX 1501 kb) [file 330_2022_8940_MOESM1_ESM.docx]

**Supplementary material**

Supplementary methods

- Automatic segmentations
- Automatic measurements
  - 3D outcomes
  - Selecting the 2D sagittal level from 3D images
  - 2D outcomes
- Correlation of MRI outcomes
- Figure S1: Correlation of MRI outcomes between t0 and t1

Table S1: Individual patient characteristics and results

Supplementary methods

**Automatic segmentations**

For 3D breath hold scans at end-inspiration and end-expiration, we developed automatic lung segmentations using convolutional neural networks. The models, based on a 3D U-Net architecture (1), were implemented and trained with the TensorFlow toolbox for Python (Python 3.6.3, https://www.python.org/, ©2001-2019. Python Software Foundation; https://www.tensorflow.org).

The training data included manual lung segmentations of 10 Pompe patients and 6 healthy controls, segmented every second axial slice using 3D Slicer (2, 3). The segmentations were interpolated to obtain a full lung segmentation that could be given to the segmentation network. The training data was extended using data augmentation with elastic deformations of the original training images (1). Before segmentation, all scans were normalized using the N4ITK bias field correction algorithm (4). Since the segmentation model did not distinguish between left and right lungs, the lungs were separated in a post-processing step. The two largest objects in the segmentation were selected as the left and right lungs, based on the position of the objects in the scan. In some scans, the left and right lungs were connected in the middle of the segmentation. In those instances, the lungs were separated by applying a watershed algorithm, based on the distance to the nearest background voxel, to find the optimal separation between both sides. After segmentation, the binary output of the network was post-processed with a morphological hole-filling operation to remove any small holes from the automatic lung segmentations. All segmentations produced by the automated models were manually checked for inaccuracies. Small adjustments were made for 4 of the 80 3D expiration segmentations, for 15 of the 80 of the 3D inspiration segmentations, mainly to correct oversegmentations or due to scanning artefacts.

**Automatic measurements**

*3D outcomes*

The 3D analysis was executed in MATLAB (Mathworks, Natick, MA). The automatic voxelwise segmentation of the lungs was converted into a 3D mesh grid of the lung surface. The surface mesh of each lung was subdivided into segments corresponding to the diaphragm and the lateral, anterior, posterior, superior and medial sides of the lung. This subdivision was made using a random walk algorithm and based on previous work (5). Based on the subdivided lung mesh at inspiration and expiration, the volume between the inspiration and expiration diaphragm was computed and used as a measure of the volume displaced by the diaphragm. This can be compared with the difference between the full lung volumes at inspiration and expiration to compute the diaphragm volume ratio.

*Selecting the 2D sagittal level from 3D images*

To perform 2D measurements in a corresponding sagittal level, sagittal slices were derived from 3D MRIs at the sagittal level of 2D scans at the initial MRI, which was manual indicated at the mid hemi-diaphragm during the scanning procedure. To obtain these segmentations, the 3D scans from the initial and follow-up MRI were registered using elastix with rigid transformations, allowing only for a rotation and translation between the two MRIs (6). The automatic segmentations of the 3D inspiration and expiration scans of the follow-up MRI were then transformed to the coordinate space of the initial MRI.

*2D outcomes*

The segmentations were analysed with a custom-built Python script (Python 3.6.3, https://www.python.org/, ©2001-2019. Python Software Foundation; SciPy 1.1.0, https://www.scipy.org/, ©2003-2019 SciPy developers) to compute thorax- and diaphragm-related outcome measures for each frame. The algorithm first determined the general orientation of the lung, to correct for minor differences in position between patients, by fitting an ellipse to the points on the lung outline. The lung apex was then defined as the highest point on the lung outline, measured in the direction of the major axis of the ellipse. By visual inspections, we verified that this approach selected a reasonable lung apex. The algorithm then detected the anterior and posterior corners of the diaphragm. We computed the distance between each point on the lung outline and the centroid (center of gravity) of the lung surface. The diaphragm corners were defined as the points with the largest distance to the centroid and located in the lower part of the lungs. Correctly finding the diaphragm corners can be difficult, especially for patients with an irregular diaphragm shape, but we found that our approach made reasonable choices for the subjects in our study. After determining the diaphragm corners, the algorithm selected the diaphragm apex as the point on the diaphragm contour that is furthest away from the linear line between both diaphragm corners. We found that for some subjects this maximum-distance point could be quite variable between consecutive frames. We therefore used a derived diaphragm apex: we computed the relative position of the diaphragm apex along the diaphragm contour in each frame, as a percentage of the diaphragm length; we computed the median of this position all frames; and then we re-computed the diaphragm apex for each frame using this median relative distance.

The remaining measurements were all derived from the previously determined points. The cranial-caudal distance is defined as the distance between the lung apex and diaphragm apex. The anterior-posterior distance is the longest distance from the front to the back of the lung, measured along the minor axis of the ellipse. The lung area is the area of the segmentation. The diaphragm height is the perpendicular distance between the diaphragm apex and the linear line connecting both diaphragm corners. The diaphragm area is the area between the diaphragm contour and the linear line.

**Correlation of MRI outcomes over time**

To investigate the variation of MRI outcomes over time, we calculated the correlation between initial (t0) MRI outcomes and follow-up (t1) MRI outcomes using Spearman correlation (figure S1). For 3D outcomes, the correlations were >0.97 (p<0.001). In sagittal slices of 3D images, the correlations of 2D thorax-related outcomes were >0.94 (p<0.001) and the correlations of 2D diaphragm-related outcomes were >0.84 (p<0.001).

Figure S1: Correlation of MRI outcomes between t0 and t1


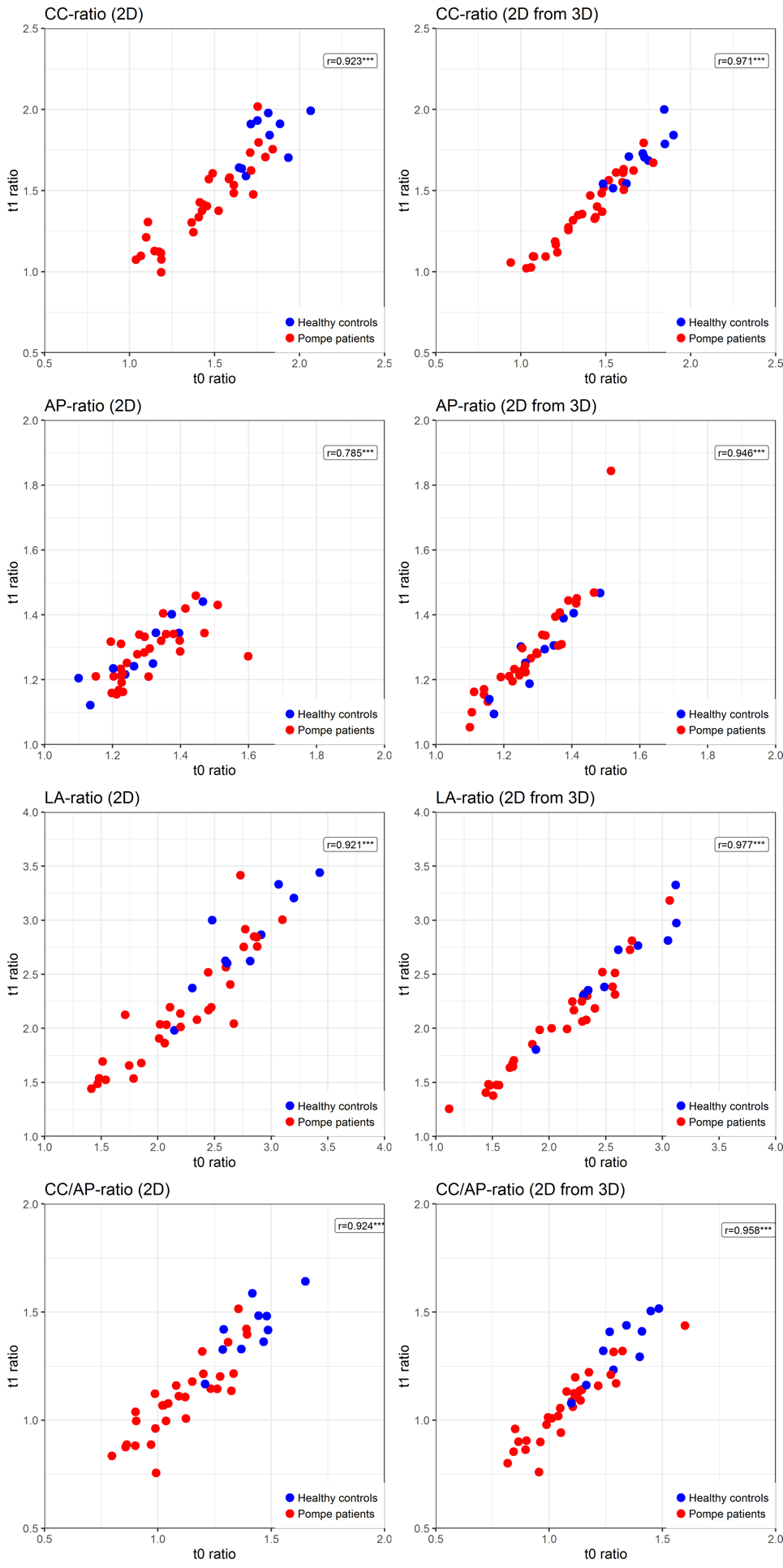

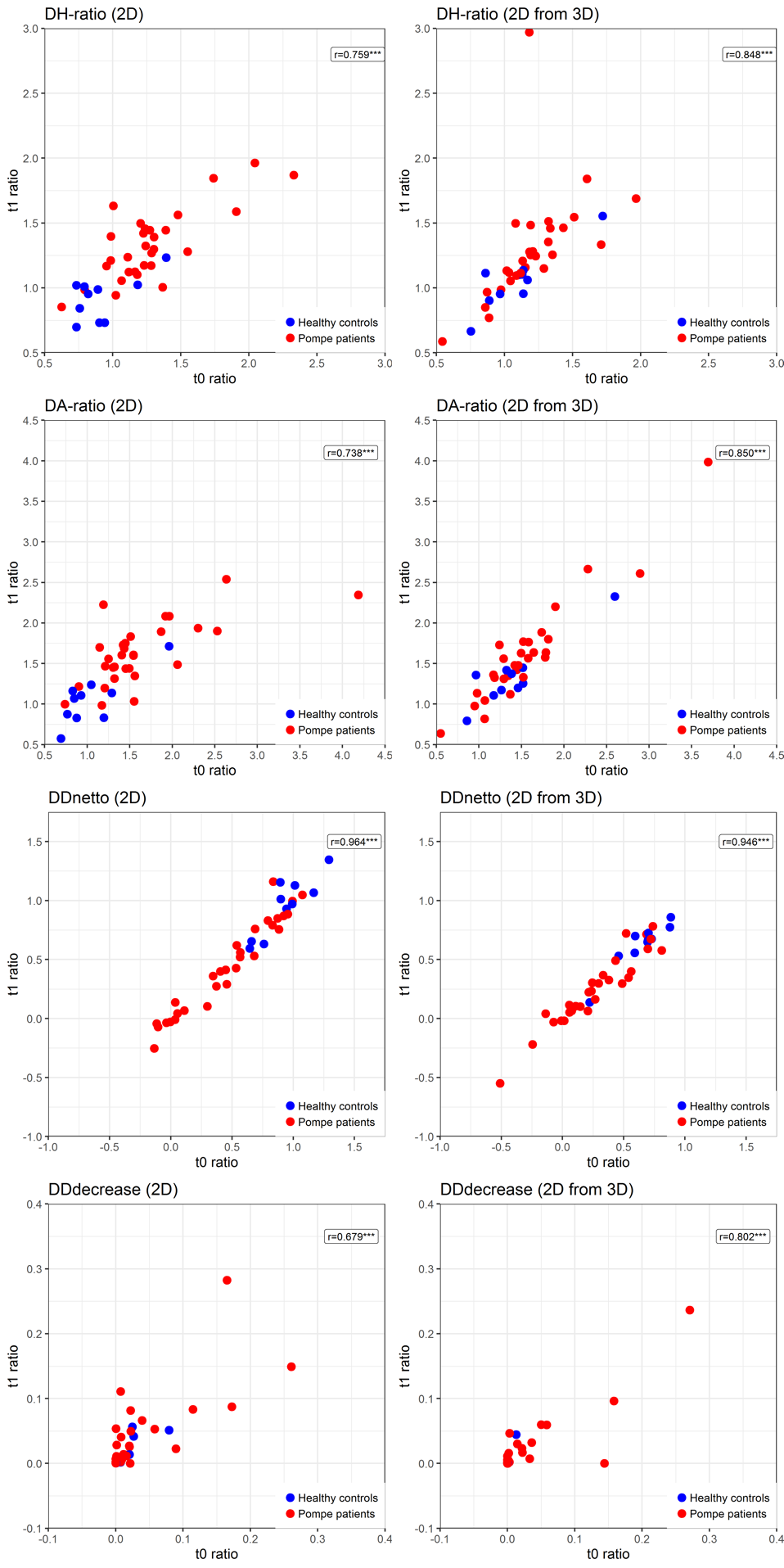

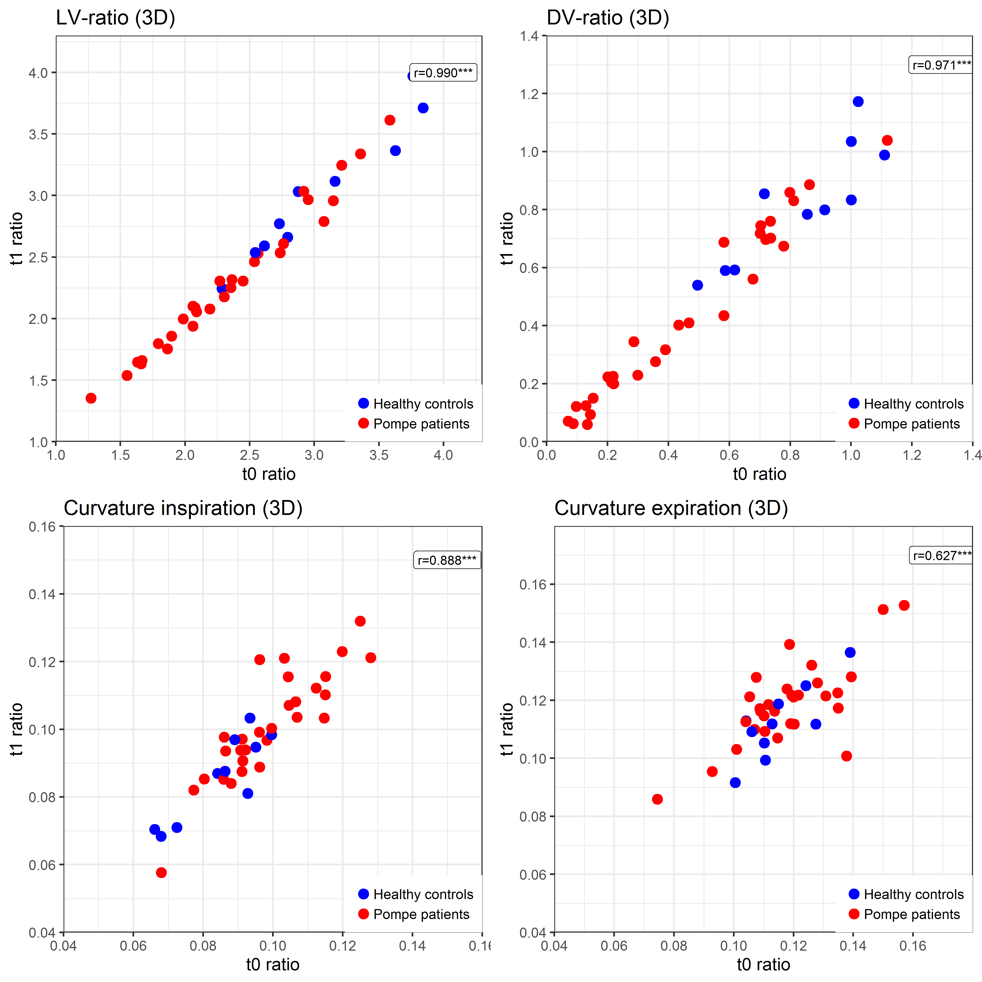

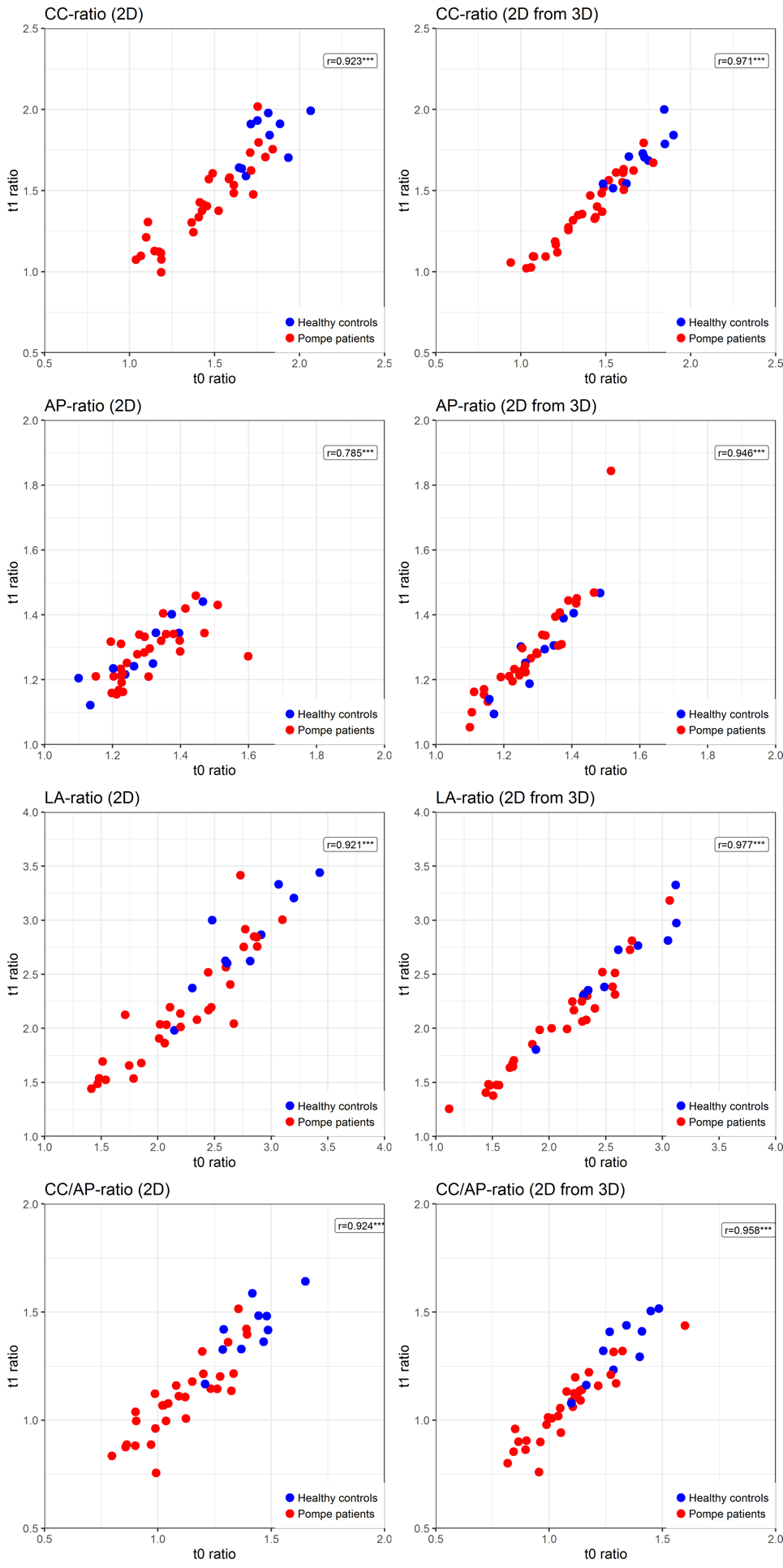

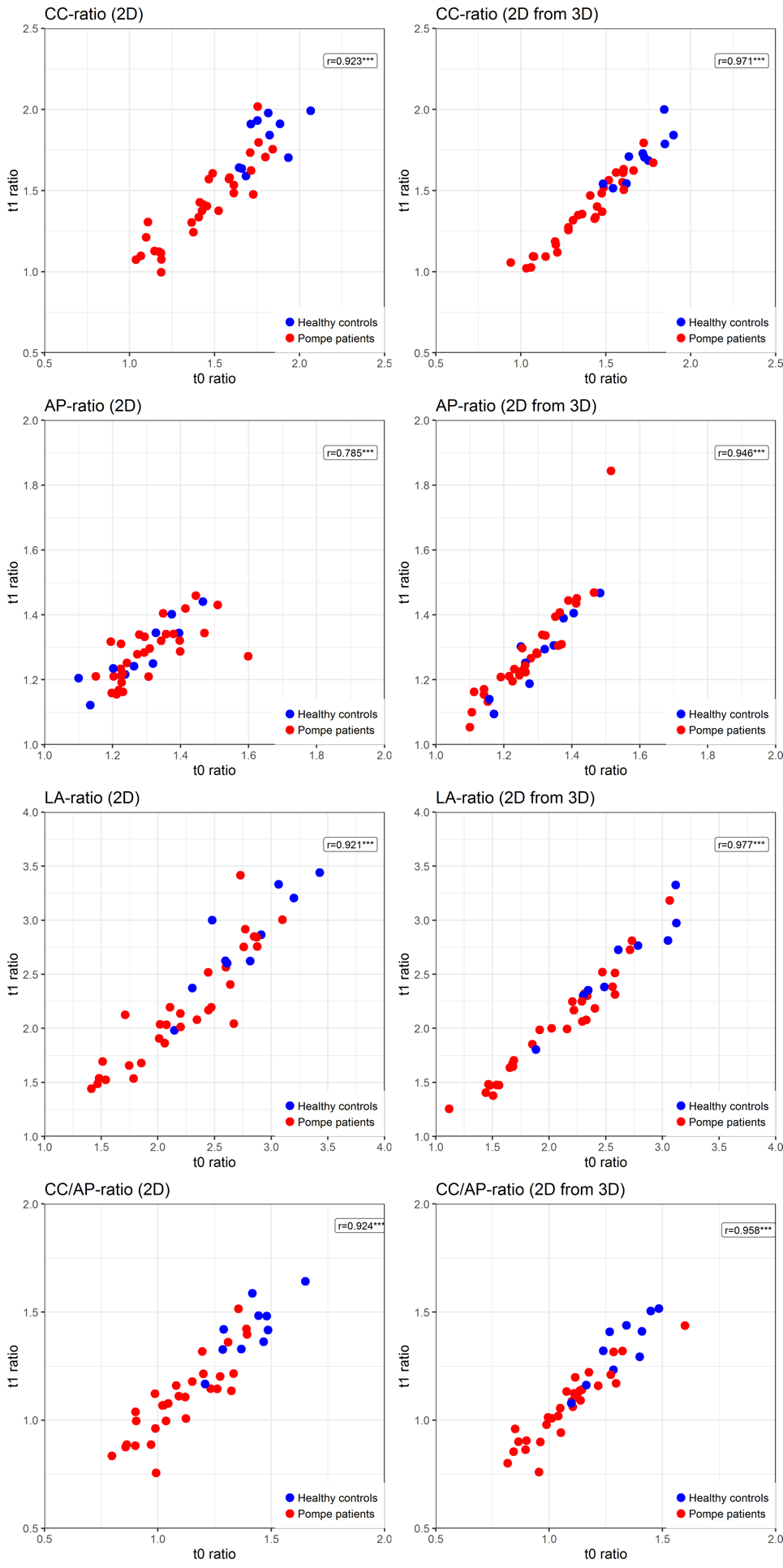

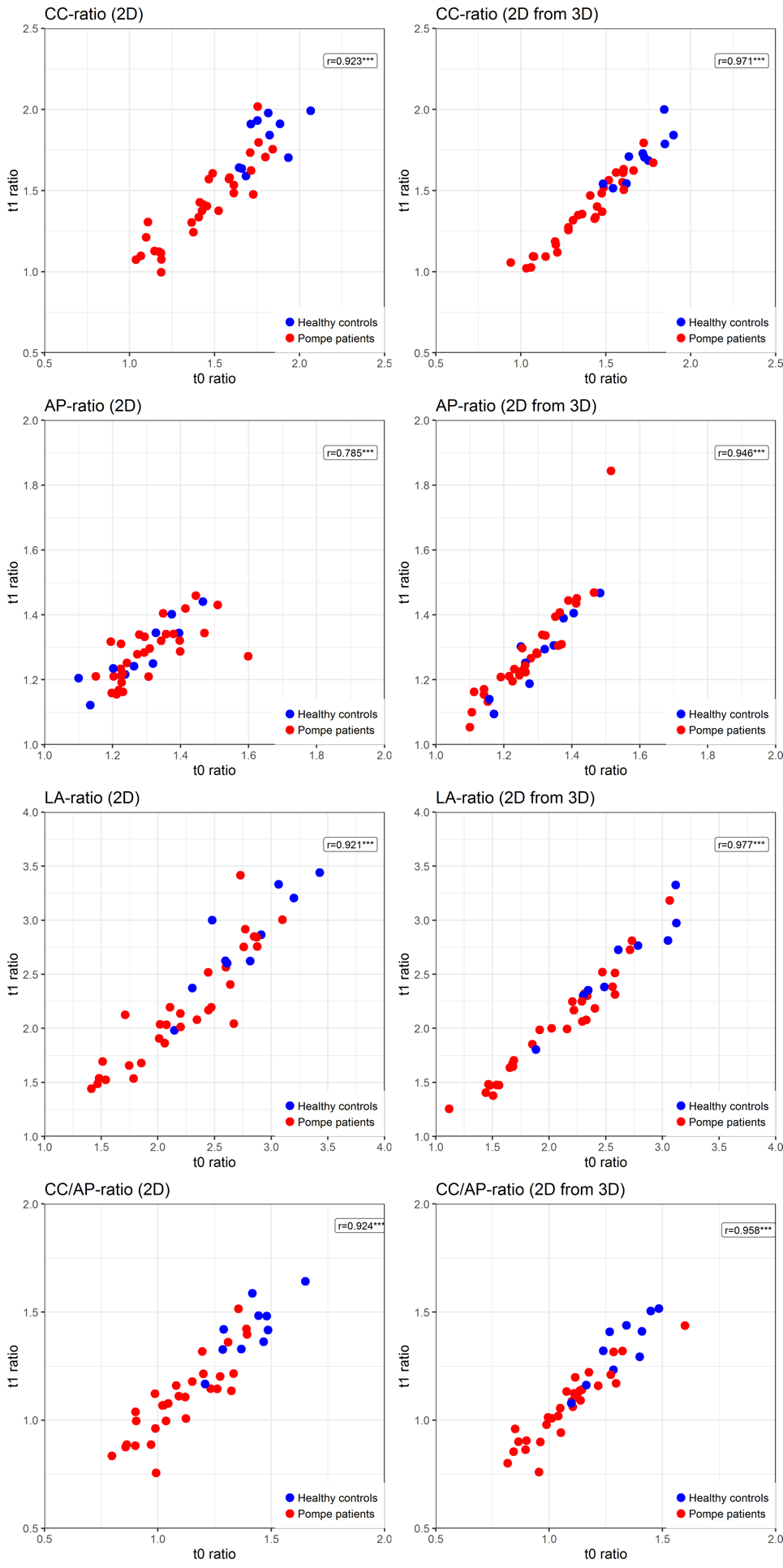

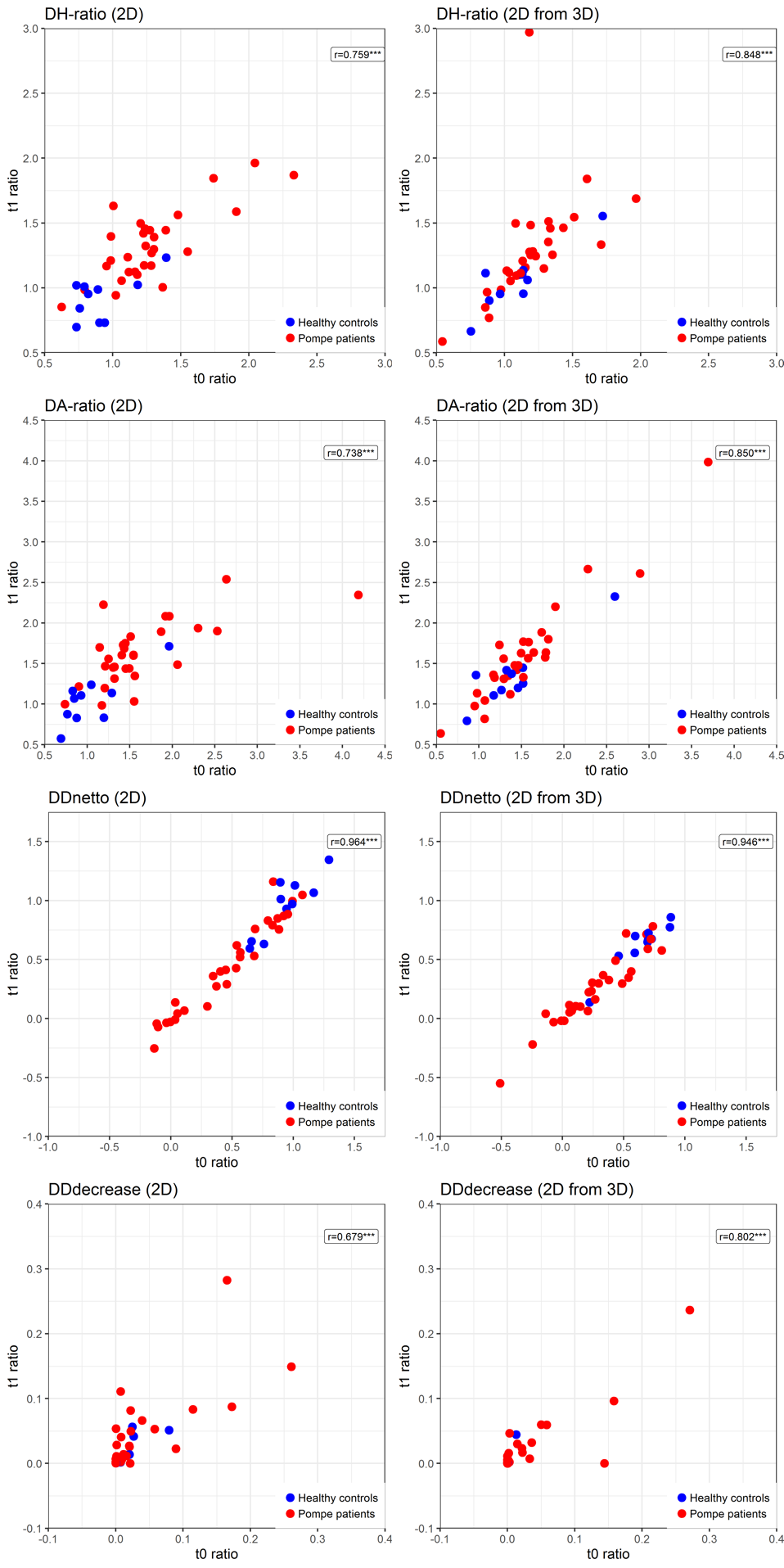


*Correlation of 3D outcomes, 2D thorax-related outcomes, 2D diaphragm-related outcomes between the measurements at the initial MRI (t0, x-axis) and follow-up MRI (t1, y-axis). LV = lung volume, DV = diaphragm volume, LA = lung area, CC = cranial-caudal, AP=anterior-posterior, DH = diaphragm height, DA = diaphragm area, r=correlation coefficient using Spearman correlation. ***=p<0.001, **=p<0.01, *=p<0.05*

**References**

1. Ronneberger O, Fischer P, Brox T. U-net: Convolutional networks for biomedical image segmentation. International Conference on Medical image computing and computer-assisted intervention: Springer, 2015; p. 234-41.

2. Wens SC, Ciet P, Perez-Rovira A, et al. Lung MRI and impairment of diaphragmatic function in Pompe disease. BMC Pulm Med. 2015;15:54.

3. Fedorov A, Beichel R, Kalpathy-Cramer J, et al. 3D Slicer as an image computing platform for the Quantitative Imaging Network. Magn Reson Imaging. 2012;30(9):1323-41.

4. Tustison NJ, Avants BB, Cook PA, et al. N4ITK: improved N3 bias correction. IEEE Trans Med Imaging. 2010;29(6):1310-20.

5. Mogalle K, Perez-Rovira A, Ciet P, et al. Quantification of Diaphragm Mechanics in Pompe Disease Using Dynamic 3D MRI. PLoS One. 2016;11(7):e0158912.

6. Klein S, Staring M, Murphy K, Viergever MA, Pluim JP. elastix: a toolbox for intensity-based medical image registration. IEEE Trans Med Imaging. 2010;29(1):196-205.

Table S1: Individual patient characteristics and results

| **Baseline characteristics** | | | | | | | | | | **Baseline test results** | | | | | | **Follow-up test results** | | | | | | |
| --- | --- | --- | --- | --- | --- | --- | --- | --- | --- | --- | --- | --- | --- | --- | --- | --- | --- | --- | --- | --- | --- | --- |
| Subject | Sex | Age (years) | Height (cm) | Weight (kg) | BMI (kg/m^2^) | Disease duration (years) | Venti-lation | Wheel-chair | ERT duration (years) | FVC upright (%pred) | FVC supine (%pred) | CC ratio (I/E) | CC-AP ratio (I/E) | DH ratio (I/E) | DA ratio (I/E) | Follow-up time (years) | FVC upright (%pred) | FVC supine (%pred) | CC ratio (I/E) | CC-AP ratio (I/E) | DH ratio (I/E) | DA ratio (I/E) |
| *Healthy controls* | | | | | | | | | | | | | | | | | | | | | | |
| HC01 | M | 32 | 194 | 90 | 24 | N/A | No | No | N/A | 105 | 105 | 1,9 | 1,41 | 1,14 | 1,46 | 1,14 | 103 | 102 | 1,84 | 1,41 | 0,96 | 1,2 |
| HC02 | M | 26 | 193 | 85 | 23 | N/A | No | No | N/A | 114 | 106 | 1,84 | 1,34 | 0,86 | 0,97 | 1 | 112 | 108 | 2 | 1,44 | 1,11 | 1,36 |
| HC03 | M | 61 | 191 | 86 | 24 | N/A | No | No | N/A | 114 | 114 | 1,85 | 1,45 | 0,75 | 0,86 | 1,41 | 109 | 107 | 1,79 | 1,51 | 0,67 | 0,79 |
| HC04 | M | 28 | 186 | 76 | 22 | N/A | No | No | N/A | 109 | 103 | 1,64 | 1,24 | 1,17 | 1,52 | 1,28 | 106 | 102 | 1,71 | 1,32 | 1,06 | 1,26 |
| HC05 | M | 40 | 194 | 99 | 26 | N/A | No | No | N/A | 99 | 96 | 1,75 | 1,4 | 1,1 | 1,33 | 1,51 | 97 | 90 | 1,69 | 1,29 | 1,1 | 1,42 |
| HC06 | F | 29 | 167 | 60 | 22 | N/A | No | No | N/A | 110 | 105 | 1,73 | 1,16 | 1,72 | 2,6 | 1,04 | 110 | 108 | 1,71 | 1,16 | 1,55 | 2,33 |
| HC07 | F | 44 | 168 | 73 | 26 | N/A | No | No | N/A | 98 | 94 | 1,72 | 1,48 | 0,89 | 1,17 | 1,07 | 94 | 91 | 1,73 | 1,52 | 0,9 | 1,11 |
| HC08 | F | 42 | 173 | 70 | 23 | N/A | No | No | N/A | 99 | 95 | 1,62 | 1,28 | 0,97 | 1,26 | 1,63 | 99 | 94 | 1,54 | 1,23 | 0,95 | 1,17 |
| HC09 | F | 25 | 165 | 55 | 20 | N/A | No | No | N/A | 96 | 93 | 1,54 | 1,1 | 1,14 | 1,52 | 1,11 | 95 | 89 | 1,52 | 1,08 | 1,14 | 1,45 |
| HC10 | F | 62 | 168 | 75 | 27 | N/A | No | No | N/A | 107 | 105 | 1,48 | 1,27 | 1,13 | 1,38 | 1,07 | 106 | 97 | 1,54 | 1,41 | 1,1 | 1,37 |
| *Pompe patients without ERT* | | | | | | | | | | | | | | | | | | | | | | |
| PP01 | M | 23 | 188 | 76 | 22 | 0 | No | No | N/A | 92 | 87 | 1,52 | 1,12 | 1,09 | 1,29 | 1,03 | 91 | 85 | 1,56 | 1,2 | 1,1 | 1,31 |
| PP02 | M | 21 | 180 | 83 | 26 | 0 | No | No | N/A | 102 | 93 | 1,66 | 1,32 | 1,34 | 1,74 | 1,01 | 96 | 90 | 1,62 | 1,32 | 1,46 | 1,89 |
| PP03 | F | 30 | 174 | 67 | 22 | 0 | No | No | N/A | 100 | 97 | 1,48 | 1,08 | 1,18 | 1,64 | 0,98 | 100 | 95 | 1,48 | 1,13 | 1,28 | 1,64 |
| PP04 | F | 34 | 172 | 62 | 21 | 0 | No | No | N/A | 90 | 69 | 1,21 | 0,96 | 1,04 | 1,18 | 0,98 | 88 | 65 | 1,17 | 0,9 | 1,05 | 1,33 |
| PP05 | F | 33 | 168 | 64 | 23 | 3,85 | No | No | N/A | 89 | 90 | 1,61 | 1,27 | 0,54 | 0,55 | 1 | 84 | 83 | 1,51 | 1,21 | 0,59 | 0,64 |
| PP06 | F | 55 | 172 | 65 | 22 | 30,1 | No | No | N/A | 110 | 102 | 1,78 | 1,6 | 0,86 | 0,95 | 1 | 99 | 92 | 1,67 | 1,44 | 0,85 | 0,98 |
| PP07 | F | 17 | 163 | 56 | 19 | 0 | No | No | N/A | 93 | 95 | 1,6 | 1,14 | 1,61 | 2,28 | 2,01 | 94 | 89 | 1,63 | 1,14 | 1,84 | 2,67 |
| *Pompe patients ≤ 3 years ERT* | | | | | | | | | | | | | | | | | | | | | | |
| PP08 | M | 56 | 175 | 84 | 27 | 7,16 | No | No | 0,07 | 92 | 84 | 1,6 | 1,28 | 0,89 | 1,07 | 0,98 | 85 | 83 | 1,61 | 1,32 | 0,77 | 0,82 |
| PP09 | M | 61 | 187 | 97 | 28 | 10,96 | No | No | -0,07 | 67 | 29 | 1,06 | 0,82 | 1,23 | 1,42 | 1 | 62 | 33 | 1,03 | 0,8 | 1,25 | 1,48 |
| PP10 | F | 70 | 168 | 58 | 21 | 13,74 | No | No | 2,95 | 79 | 62 | 1,03 | 0,84 | 1,43 | 1,45 | 1,13 | 69 | 61 | 1,02 | 0,85 | 1,46 | 1,42 |
| PP11 | F | 25 | 176 | 84 | 27 | 3,84 | No | No | 2,53 | 119 | 115 | 1,72 | 1,18 | 1,97 | 2,89 | 0,96 | 115 | 113 | 1,79 | 1,22 | 1,69 | 2,61 |
| PP12 | F | 55 | 167 | 62 | 22 | 6,89 | No | No | -0,03 | 104 | 70 | 1,2 | 0,99 | 1,32 | 1,58 | 1,03 | 97 | 70 | 1,19 | 0,98 | 1,35 | 1,57 |
| PP13 | F | 43 | 172 | 63 | 21 | 11,07 | No | No | -0,02 | 79 | 66 | 1,43 | 1,05 | 1,51 | 1,9 | 0,88 | 76 | 63 | 1,33 | 0,94 | 1,55 | 2,2 |
| PP14 | F | 36 | 166 | 71 | 26 | 11,02 | No | No | 1,23 | 73 | 47 | 0,94 | 0,85 | 1,12 | 1,37 | 1,1 | 72 | 42 | 1,06 | 0,96 | 1,11 | 1,12 |
| PP15 | F | 42 | 177 | 70 | 22 | 26,12 | No | No | 1,83 | 96 | 72 | 1,36 | 1,05 | 1,13 | 1,46 | 1,28 | 96 | 76 | 1,36 | 1,06 | 1,21 | 1,48 |
| *Pompe patients > 3 years ERT* | | | | | | | | | | | | | | | | | | | | | | |
| PP16 | M | 70 | 197 | 80 | 21 | 26,7 | Yes | No | 7,04 | 92 | 54 | 1,15 | 0,9 | 1,02 | 1,17 | 1,08 | 71 | 42 | 1,09 | 0,86 | 1,13 | 1,36 |
| PP17 | M | 38 | 187 | 85 | 24 | 37,72 | No | No | 8,09 | 69 | 53 | 1,31 | 1,14 | 0,87 | 0,98 | 1,13 | 69 | 50 | 1,32 | 1,14 | 0,97 | 1,14 |
| PP18 | M | 35 | 178 | 93 | 29 | 11,61 | No | No | 3,67 | 90 | 89 | 1,6 | 1,22 | 1,19 | 1,82 | 0,98 | 88 | 85 | 1,55 | 1,16 | 1,25 | 1,8 |
| PP19 | M | 63 | 173 | 64 | 21 | 20,83 | No | No | 9,33 | 91 | 54 | 1,08 | 0,86 | 1,15 | 1,35 | 1 | 81 | 53 | 1,09 | 0,9 | 1,16 | 1,35 |
| PP20 | M | 18 | 186 | 69 | 20 | 13,62 | No | No | 7,66 | 100 | 98 | 1,41 | 1 | 1,35 | 1,52 | 1,94 | 104 | 98 | 1,47 | 1,01 | 1,26 | 1,33 |
| PP21 | M | 25 | 175 | 69 | 23 | 11,04 | No | No | 10,63 | 98 | 98 | 1,56 | 1,12 | 1,29 | 1,79 | 1 | 98 | 95 | 1,61 | 1,12 | 1,15 | 1,64 |
| PP22 | M | 48 | 185 | 79 | 23 | 17,16 | No | No | 8,68 | 95 | 74 | 1,28 | 1,11 | 0,98 | 1,07 | 1 | 92 | 72 | 1,27 | 1,12 | 0,99 | 1,04 |
| PP23 | M | 24 | 195 | 86 | 23 | 11,44 | No | No | 10,16 | 79 | 65 | 1,21 | 1,1 | 1,19 | 1,29 | 1,71 | 68 | 50 | 1,12 | 1,06 | 1,48 | 1,56 |
| PP24 | M | 42 | 172 | 95 | 32 | 20,35 | No | No | 11,18 | 70 | 48 | 1,45 | 0,96 | 1,18 | 1,67 | 1,19 | 66 | 48 | 1,4 | 0,76 | 2,97 | 6,07 |
| PP25 | M | 18 | 201 | 99 | 25 | 16,04 | No | No | 12,78 | 82 | 72 | 1,49 | 1,1 | 1,03 | 1,52 | 1,05 | 77 | 72 | 1,52 | 1,09 | 1,12 | 1,77 |
| PP26 | F | 45 | 169 | 78 | 27 | 11,77 | No | No | 7,03 | 81 | 59 | 1,28 | 1,04 | 1,32 | 1,58 | 1 | 81 | 59 | 1,26 | 1,02 | 1,51 | 1,76 |
| PP27 | F | 67 | 168 | 64 | 23 | 19,02 | No | No | 9,21 | 68 | 41 | 1,07 | 0,9 | 1,71 | 1,78 | 1 | 71 | 42 | 1,09 | 0,91 | 1,33 | 1,58 |
| PP28 | F | 48 | 157 | 58 | 24 | 16,62 | No | No | 9,14 | 97 | 87 | 1,44 | 1,14 | 1,08 | 1,24 | 1,02 | 96 | 87 | 1,34 | 1,09 | 1,5 | 1,73 |
| PP29 | F | 54 | 173 | 69 | 23 | 18,71 | No | No | 9,87 | 89 | 67 | 1,34 | 1,01 | 2,78 | 3,69 | 1,05 | 80 | 64 | 1,35 | 1,01 | 2,82 | 3,99 |
| PP30 | F | 45 | 163 | 89 | 34 | 13,03 | No | No | 10,5 | 86 | 68 | 1,48 | 1,29 | 1,21 | 1,49 | 1,05 | 86 | 68 | 1,37 | 1,17 | 1,28 | 1,63 |

*BMI = body mass index, CC ratio = cranial-caudal ratio, CC-AP = cranial-caudal / anterior-posterior ratio, DH ratio = diaphragm height ratio, DA ratio = diaphragm area ratio, ERT = enzyme replacement therapy, F = female, FVC = forced vital capacity, HC = healthy control, I/E = inspiration / expiration, M = male, N/A = not applicable, PP = Pompe patient, %pred = % of predicted value*
